# A mobile app compared with an educational pamphlet on menopause hormone therapy initiation: a randomized pilot study

**DOI:** 10.3389/frph.2026.1952673

**Published:** 2026-09-16

**Authors:** Juliana M. Kling, Courtney A. French, Ekta Kapoor, Mary S. Hedges, Margaret M. Paul, Yihong Deng, Rajeev Chaudhry, Jamie L. Smith, Regina Castaneda, Kristin C. Cole, Chrisandra L. Shufelt, Arjan Karens, Stephanie S. Faubion

**Affiliations:** 1 Division of Women’s Health Internal Medicine, Mayo Clinic, Scottsdale, AZ, United States; 2 Mayo Clinic Center for Women’s Health, Mayo Clinic, Rochester, MN, United States; 3 University of Notre Dame, South Bend, IN, United States; 4 Women’s Health Research Center, Mayo Clinic, Rochester, MN, United States; 5 Division of Endocrinology, Diabetes, Metabolism, and Nutrition, Mayo Clinic, Rochester, MN, United States; 6 Department of Medicine, Division of Community Internal Medicine, Jacksonville, FL, United States; 7 Robert D. and Patricia E. Kern Center for the Science of Health Care Delivery, Mayo Clinic, Rochester, MN, United States; 8 Robert D. and Patricia E. Kern Center for the Science of Health Care Delivery, Mayo Clinic, Scottsdale, AZ, United States; 9 Division of Community Internal Medicine, Mayo Clinic Health System, Eau Claire, WI, United States; 10 Division of General Internal Medicine, Mayo Clinic, Jacksonville, FL, United States; 11 Department of Quantitative Health Sciences, Mayo Clinic, Rochester, MN, United States; 12 BettrHealth, Ede, Netherlands

**Keywords:** digital tool, education, health application, menopause, menopause education

## Abstract

**Background:**

Menopause-related symptoms are common but undertreated. Digital tools that support symptom assessment and patient education may improve engagement.

**Objective:**

To assess whether a previsit digital menopause symptom assessment and education tool increased treatment initiation and patient engagement compared to an educational pamphlet.

**Design:**

Women aged 45–55 years with upcoming primary care appointments were invited to participate. Those with a total Menopause Rating Scale (MRS) score ≥5 indicating bothersome menopause symptoms were included. Participants were randomized to a digital menopause symptom assessment tool or a digital educational pamphlet and completed a post-visit survey (NCT06919887).

**Participants:**

Of the 14,726 women invited to participate between May 12, 2025, and December 5, 2025, there were 653 (4.4%) enrolled that met eligibility criteria.

**Interventions:**

Randomized to an mHealth app called emmii or a standard digital menopause educational pamphlet.

**Main measures:**

Menopause treatment initiation within 30 days of the clinical visit was assessed by electronic health data. Baseline and post-visit surveys were collected assessing patient experience, engagement, and perceived usefulness. Rates of menopause hormone therapy (HT) prescribing were compared with a reference population of nonparticipants aged 45–55 who had a primary care appointment at Mayo Clinic during the same period and received no study education materials.

**Key results:**

Of 653 participants enrolled and randomized, 383 completed both the baseline and post-surveys (171 intervention and 212 control). HT initiation among study participants was significantly higher than in the reference population of women (9.3% vs. 4.4%, *p* < 0.001). There was no difference in HT initiation between the intervention (9.1%) and control groups (9.6%) (*p* = 0.83). Initiation rates were 3.8% and 4.5%, respectively (*p* = 0.64) for nonhormone therapies.

**Conclusions:**

Among women with bothersome menopause symptoms, those who underwent previsit symptom screening and received educational materials had higher rates of HT initiation than women who did not participate in the study. These findings suggest that structured menopause education may improve recognition and management of menopause symptoms in routine clinical practice.

## Introduction

Menopause is diagnosed retrospectively after 12 consecutive months of amenorrhea (1). During perimenopause and menopause, approximately 50%–75% of women experience vasomotor symptoms, 45%–68% experience psychological symptoms, and more than 50% develop genitourinary symptoms (2, 3). Menopause hormone therapy (HT) is the first-line treatment for bothersome vasomotor and genitourinary symptoms in symptomatic women without contraindications at and around the time of menopause. HT may also alleviate menopause-related sleep and psychological symptoms (2, 3). For women with contraindications to HT or a personal preference not to use it, multiple effective nonhormonal treatments and lifestyle interventions exist (4).

Barriers to menopause treatment exist in clinical settings. Women may hesitate to report menopause symptoms due to prior negative experiences, discomfort discussing menopause, or due to the belief that primary care is not the appropriate setting to address such concerns (5, 6). Furthermore, clinicians often lack the training, confidence, or time to routinely screen for and manage menopause-related symptoms (7, 8). Identifying solutions to these barriers could help to address treatment gaps, including with emerging technologies such as mobile health (mHealth) apps.

With the widespread use of smartphones, mHealth apps have emerged as useful tools for health care management (9). These apps offer a range of functions, from educational resources and symptom tracking to medical record access and therapeutic guidance, with an estimated one app for every medical condition (10, 11). Research suggests that menstrual health and cognitive behavioral therapy apps can enhance physical and psychological well-being (12, 13). A small, but growing, number of mHealth apps focus specifically on menopause, addressing patient education, symptom tracking, and therapeutic guidance (11). A longitudinal study of 1,900 women using the “Health & Her” app found that those who engaged more frequently with the app's features such as symptom tracking, period logging, and using in-app activities experienced significantly greater reductions in menopause symptoms over a 2-month period (14). Women randomized to use Caria, an mHealth app for menopause with an artificial intelligence chatbot-assisted symptom assessment and a 6-week self-guided program with daily cognitive behavioral therapy, mindfulness-based exercises, physical activity video sessions, and community-based peer support had significantly reduced distress related to hot flashes compared to controls with no difference in the frequency of hot flashes (15). The app did not directly facilitate engagement with clinicians or support shared decision-making about medication management.

Despite their potential benefits, most available menopause mHealth apps do not explicitly support shared decision-making or used evidence-based tools to address women's individual needs. Key gaps in menopause mHealth apps include menopause knowledge deficits, lack of guidance on treatment options, limited shared decision-making support, and challenges in symptom self-management. A 2019 systematic review of 22 mHealth menopause apps found that only 27.3% of the apps involved medical professionals in their development, and only 22.7% used evidence-based information, raising concerns about the quality and reliability of these tools (11). As a result, many women remain underinformed about available therapies or uncertain about how to engage with clinicians regarding their menopause symptoms and treatment preferences.

To address these gaps, the Mayo Clinic Center for Women's Health, in collaboration with a digital health company, BettrHealth, codeveloped a patient-centered mHealth app called emmii for menopause symptom identification and management. The app was designed to provide evidence-based menopause education, individualized symptom assessment, treatment guidance, and a printable discussion guide to facilitate shared decision-making in the primary care setting. This study evaluated whether the app increased new HT initiation in a clinical setting compared with a standard menopause educational pamphlet.

## Methods

### Design and participants

This study was a randomized controlled trial evaluating the emmii app vs. an educational pamphlet in women aged 45–55 years of age with a primary care appointment within three weeks of enrollment at any of the following Mayo Clinic locations (Arizona, Florida, Minnesota, or Wisconsin), between May 12, 2025 and December 5, 2025 (NCT06919887). Participants that met this inclusion criteria were identified through the electronic health record (EHR) and sent a link via email inviting them to complete the Menopause Rating Scale (MRS) and the informed consent form. Women who had a total MRS score of ≥5, indicating bothersome menopause symptoms, were randomized to either the intervention group or the control group. Participants were randomly assigned to one of two groups in a 1:1 allocation using a computer-generated random number generator. The intervention group received access to the emmii app, and the control group digitally received a standard menopause educational pamphlet. The materials were provided to participants before their primary care visits. After the visit, participants were asked to complete a post-visit survey about their experiences. The pamphlet included educational content on perimenopause and menopause symptoms, hormone and nonhormone treatment options, and lifestyle strategies based on clinical guidelines. Although it offered similar content to the app, it did not include emmii's interactive or personalized features (Supplementary Appendix 1). Patients who were unable to provide consent or were unwilling to engage with the app were excluded from the study. Participants provided informed consent prior to taking part in the study which was approved by the Mayo Clinic Institutional Review Board (IRB ID# 24-013662) (Supplementary Appendix 2).

#### Post-visit survey

A post-visit survey was distributed 1–3 weeks after the participant's primary care visit to assess their use of the app or pamphlet. The post-visit survey included quantitative questions with 65 discrete questions for those who received the app and 54 discrete questions for those who received the pamphlet. The additional questions for those who received the app were related to satisfaction with the app. Participants rated how important common menopause-related symptoms, on a scale from 1 (not at all important) to 5 (extremely important), are to their current healthcare needs. In addition, the survey assessed whether menopause symptoms were discussed during the clinic visit, their priorities for menopause-related health care, their perceived access to menopause care, and preferences for receiving information on menopause symptoms and treatment options. Women received up to 3 reminder messages and had up to 3 weeks after their appointments to complete the survey.

Clinical outcomes were assessed based on the use of HT at baseline and 30 days following the primary care visit. HT initiation was defined as a new estrogen prescription order within 30 days after the appointment date and with a 1-year washout period with no qualifying estrogen orders. The current definition includes both systemic and vaginal estrogen formulations.

To assess the effect of menopause education more broadly, rates of HT prescribing were compared between the combined study population (intervention and control groups) and a reference population comprising women aged 45–55 who had a primary care appointment at Mayo Clinic during the same study period but did not participate in this study nor receive menopause education materials (16).

### App development and beta testing

The emmii app was codeveloped through a collaboration between the Mayo Clinic Center for Women's Health and BettrHealth, a digital health company, to offer comprehensive menopause education, evidence-based treatment options, and a personalized discussion guide for patients. The app then underwent beta testing where a convenience sample of 24 women aged 40 years and older were recruited by email to test the app for usability (16).

### Statistical analysis

Descriptive statistics were calculated for both clinical outcomes and survey responses, including frequencies and percentages for categorical variables, and means and standard deviations (SD) for continuous variables. Chi-square (*χ*^2^) tests of independence were used to assess associations between categorical variables. Independent sample *t*-tests were conducted to compare group differences on continuous variables.

For ranking variables, response distributions were summarized as categorical frequencies and percentages. Mann–Whitney *U* tests were then conducted to compare rankings between two independent groups, given the ordinal nature of the data. Results are reported with the corresponding *p*-values. Comparisons for specific symptoms were considered exploratory, and *p*-values were not adjusted for multiple comparisons. Accordingly, findings from these analyses should be interpreted cautiously. The statistical analysis was conducted and verified independently of BettrHealth. For all analyses, a significance threshold of *p* < 0.05 was applied. All the analyses were conducted using SAS 9.04.

## Results

A total of 14,726 eligible participants who were established primary care patients at any Mayo Clinic site were identified and invited to participate. Of those, 653 (4.4%) agreed to participate, met the criteria of MRS score ≥5, and were included in the study. Both the pre-visit and post-visit surveys were completed by 383 participants who were then included in the analytic sample: 171 in the intervention group and 212 in the control group (Figure 1). The reference sample consisted of 14,032 women aged 45–55 years old who had a primary care appointment during the study period.

**Figure 1:**
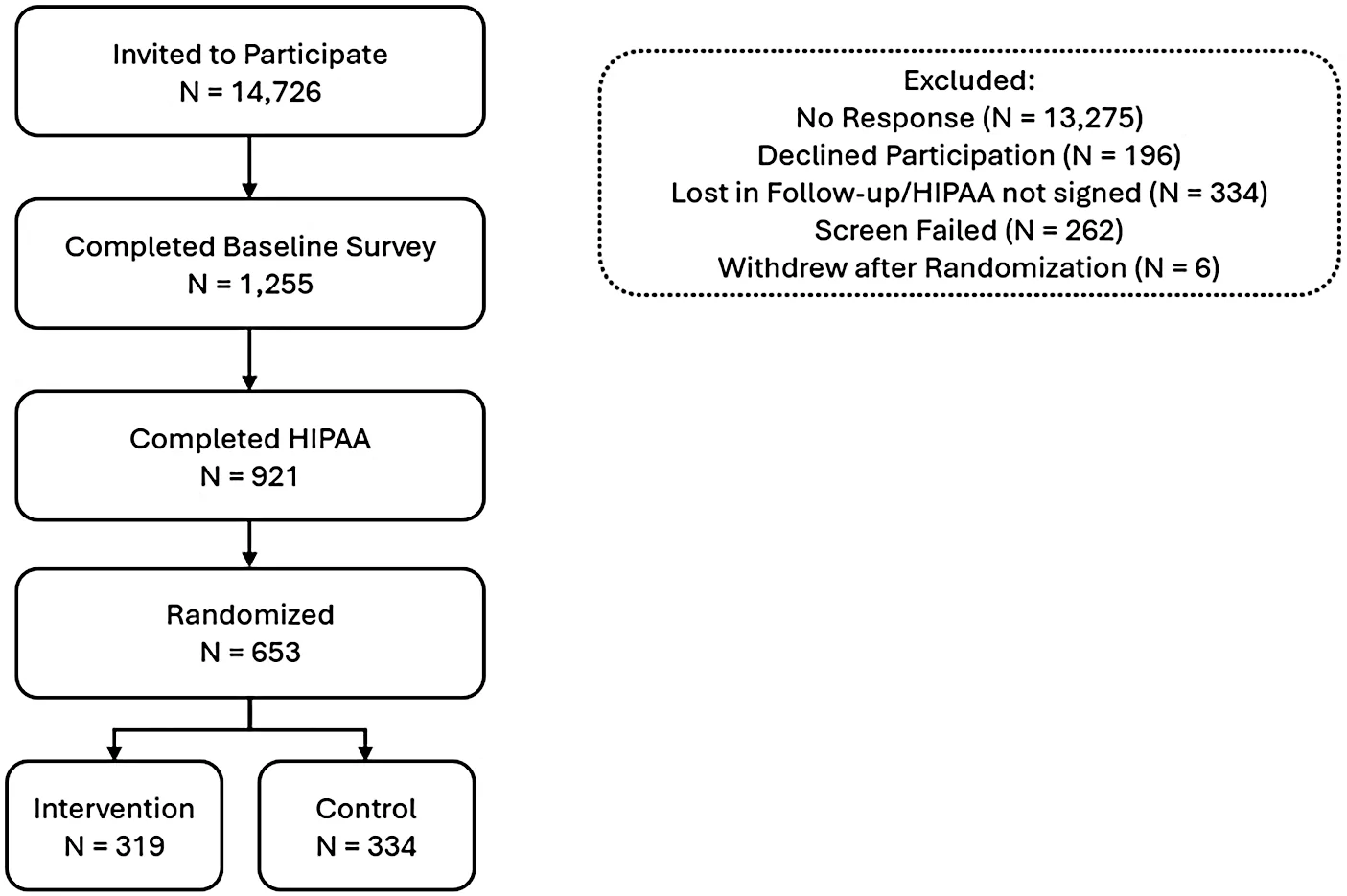
Study recruitment and participation data.

### Demographics

Mean age was 50.0 ± 3.31 years in the intervention group and 49.9 ± 3.07 years in the control group. The mean BMI was 29.4 ± 7.02 kg/m^2^ and 30.5 ± 8.43 kg/m^2^, respectively. The majority of participants in both groups identified as White (94.1% intervention group and 92.5% control group) and were not Hispanic or Latino (96.5% vs. 94.8%). Educational attainment was similarly distributed, with approximately 70% of participants in each group holding a bachelor's degree or higher (70.8% intervention, 69.7% control). Most participants carried commercial insurance (94.2% intervention, 91.0% control) (Table 1). Total MRS scores did not differ between groups (intervention: 18.0 ± 6.86; control: 17.0 ± 6.59), indicating that symptom severity was comparable.

**Table 1:** Baseline demographics survey.

Variable	Intervention (*N* = 171)	Control (*N* = 212)	Total (*N* = 383)
Age			
Mean (SD)	50.0 (3.31)	49.9 (3.07)	49.9 (3.18)
Median	50.0	50.0	50.0
Range	45.0, 55.0	45.0, 55.0	45.0, 55.0
BMI (kg/m^2^)			
Mean (SD)	29.4 (7.02)	30.5 (8.43)	30.0 (7.84)
Median	27.4	28.6	27.9
Range	17.4, 51.5	17.7, 70.2	17.4, 70.2
Ethnicity, *n* (%)			
Hispanic or Latino	3 (1.8%)	7 (3.3%)	10 (2.6%)
Not Hispanic or Latino	165 (96.5%)	201 (94.8%)	366 (95.6%)
Unknown	3 (1.8%)	4 (1.9%)	7 (1.8%)
Race, *n* (%)			
American Indian or Alaska Native	0 (0.0%)	4 (1.9%)	4 (1.0%)
Black or African American	5 (2.9%)	6 (2.8%)	11 (2.9%)
East Asian	1 (0.6%)	0 (0.0%)	1 (0.3%)
South Asian	0 (0.0%)	3 (1.4%)	3 (0.8%)
Southeast Asian	1 (0.6%)	3 (1.4%)	4 (1.0%)
White	160 (94.1%)	196 (92.5%)	356 (93.2%)
Other	3 (1.8%)	0 (0.0%)	3 (0.8%)
Missing	1	0	1
Education, *n* (%)			
High school degree or equivalent (e.g., GED)	8 (4.7%)	7 (3.3%)	15 (3.9%)
Some college, no degree	21 (12.3%)	25 (11.8%)	46 (12.0%)
Associate degree	21 (12.3%)	32 (15.2%)	53 (13.9%)
Bachelor degree	62 (36.3%)	70 (33.2%)	132 (34.6%)
Graduate degree (e.g., MA, MD, PhD)	59 (34.5%)	77 (36.5%)	136 (35.6%)
Missing	0	1	1
Income, *n* (%)			
Less than $20,000	3 (1.8%)	9 (4.2%)	12 (3.1%)
$20,000 to $34,999	4 (2.3%)	2 (0.9%)	6 (1.6%)
$35,000 to $49,999	3 (1.8%)	11 (5.2%)	14 (3.7%)
$50,000 to $74,999	17 (9.9%)	9 (4.2%)	26 (6.8%)
$75,000 to $99,999	22 (12.9%)	23 (10.8%)	45 (11.7%)
$100,000 to $149,999	43 (25.1%)	55 (25.9%)	98 (25.6%)
Over $150,000	67 (39.2%)	87 (41.0%)	154 (40.2%)
Prefer not to answer	12 (7.0%)	16 (7.5%)	28 (7.3%)
Residence state, *n* (%)			
AZ	29 (17.0%)	37 (17.5%)	66 (17.2%)
FL	34 (19.9%)	30 (14.2%)	64 (16.7%)
MN	78 (45.6%)	97 (45.8%)	175 (45.7%)
Other	3 (1.8%)	9 (4.2%)	12 (3.1%)
WI	27 (15.8%)	39 (18.4%)	66 (17.2%)
Insurance, *n* (%)			
Commercial	161 (94.2%)	193 (91.0%)	354 (92.4%)
Medicaid	6 (3.5%)	11 (5.2%)	17 (4.4%)
Medicare	2 (1.2%)	5 (2.4%)	7 (1.8%)
Other	2 (1.2%)	3 (1.4%)	5 (1.3%)
MRS score			
Mean (SD)	18.0 (6.86)	17.0 (6.59)	17.4 (6.72)
Median	17.0	17.0	17.0
Range	5.0, 37.0	5.0, 35.0	5.0, 37.0

The survey analyses below only included patients who completed both baseline and post-surveys (*N* = 383).

### Hormonal therapy initiation

The rate of new HT initiation within 30 days after the appointment was higher in the study population than in the general population [61/653 (9.34%) vs. 623/14,032 (4.44%); absolute difference, 4.90 percentage points; 95% CI: 2.64–7.16; *p* < 0.001]. Prior HT use in the year preceding the appointment was higher in the study population than in the general population [187/653 (28.64%) vs. 2,440/14,032 (17.39%); absolute difference, 11.25 percentage points; 95% CI: 7.73–14.77; *p* < 0.001]. Within the randomized study population, there was no evidence of a difference in new HT initiation within 30 days between the intervention group [29/319 (9.09%)] and the control group [32/334 (9.58%); absolute difference, −0.49 percentage points; 95% CI: −4.95 to 3.97; *p* = 0.83]. Among these participants, baseline MRS total scores were similar between those who initiated HT and those who did not (18.8 ± 7.0 vs. 17.9 ± 6.8), respectively; data not shown.

#### Post-appointment survey

In the follow-up survey, among the 383 patients who completed the survey, conditions rated as important (score of 4 or 5) by more than half of participants included weight gain and diet (70.0%), cognitive changes such as memory problems and difficulty concentrating (69.2%), sleep disturbances (62.4%), mood swings and emotional changes (60.6%), hot flashes and night sweats (55.6%), and joint pain and stiffness (54.6%) (Figure 2).

**Figure 2:**
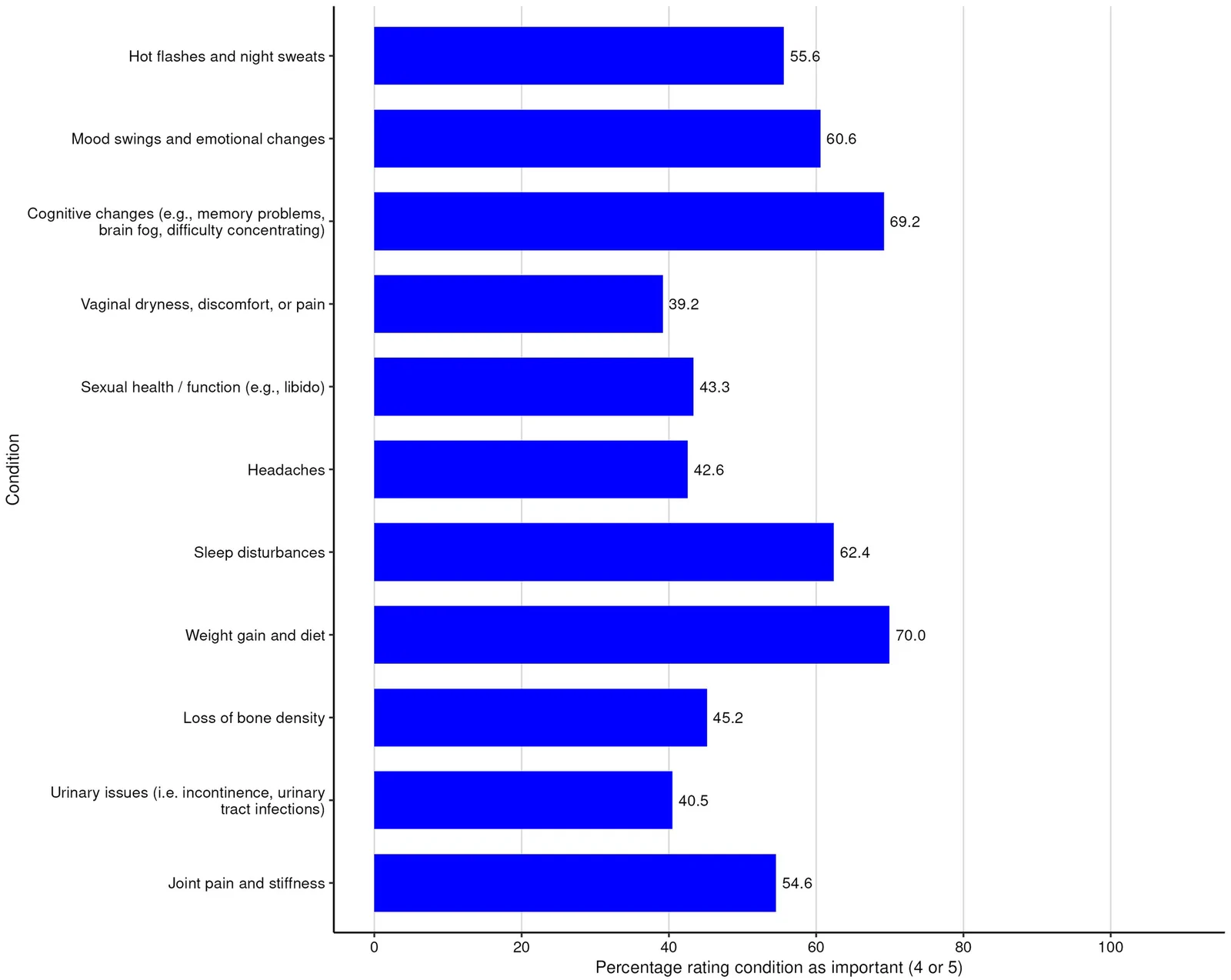
Percentage of respondents rating specific symptoms or conditions as important (4 or 5) in their healthcare needs within the next year.

Across all symptoms surveyed, satisfaction with current healthcare offerings was consistently lower than participants' corresponding importance ratings (Figure 3). For example, while 70.0% of participants rated weight gain and diet as an important healthcare need, only 35.2% were satisfied with the care they currently receive for this condition. In exploratory analyses on specific symptoms, sleep disturbances were the only symptom for which importance ratings differed between the intervention and control groups at the nominal significance threshold (unadjusted *p* = 0.04). Given that the symptom-specific analyses were not adjusted for multiple comparisons, this finding should be interpreted as exploratory. No statistically significant differences in satisfaction ratings were observed between the intervention and control groups for any symptom surveyed.

**Figure 3:**
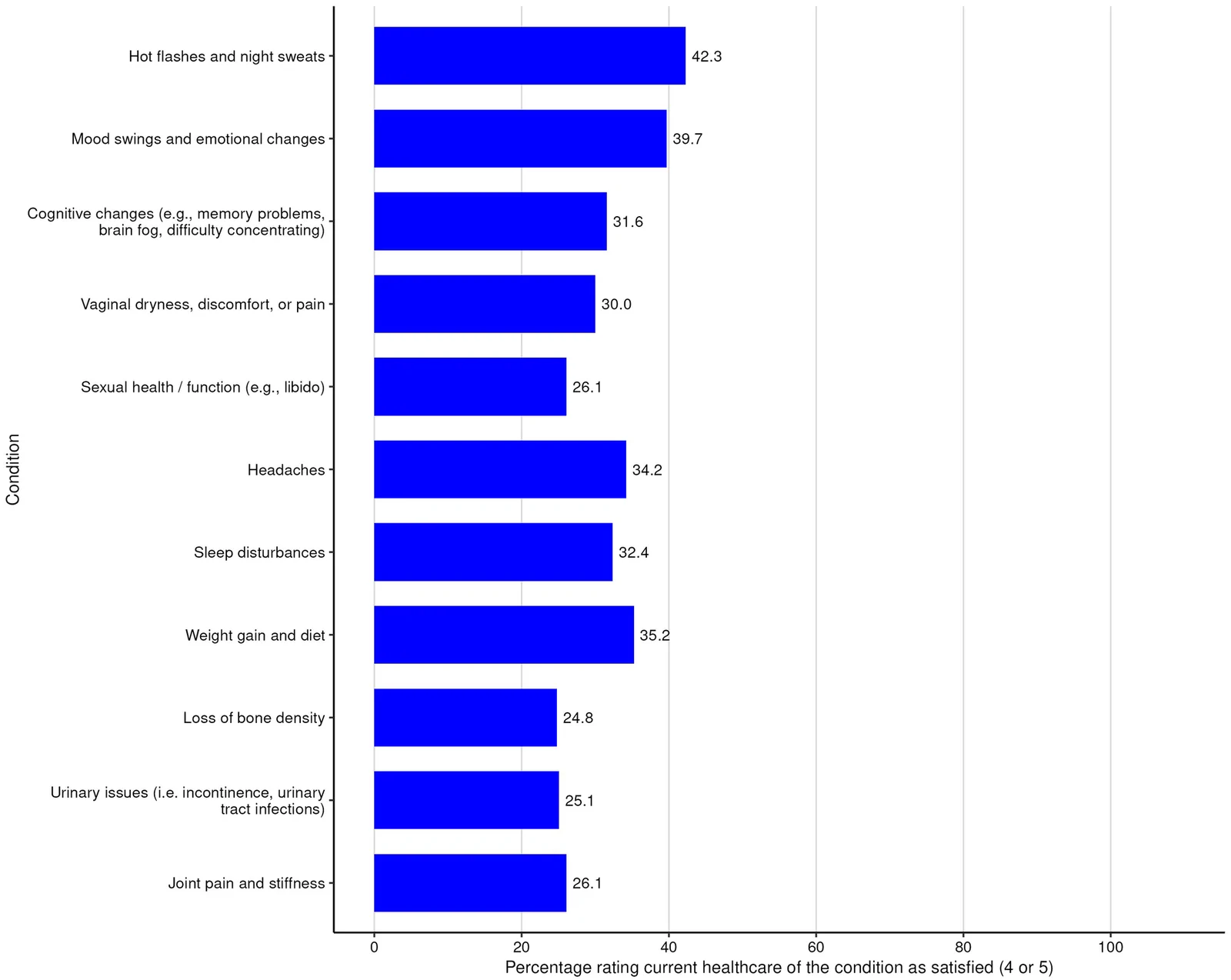
Percentage of respondents rating satisfied (4 or 5) with current healthcare offering for the specific symptom or condition.

After the intervention, 75% of users reported improved understanding of menopause symptoms, 68% reported that completing the digital tool before their appointment was useful, and more than half reported increased confidence in asking questions about and discussing symptoms related to menopause with their clinicians after using emmii. The control group was not asked these additional questions in the post-visit survey.

## Discussion

In this pilot study, receiving menopause education through either the emmii app or an educational pamphlet was associated with a higher rate of HT initiation compared with women in the reference population. This finding suggests that access to menopause education, regardless of delivery format, may meaningfully increase HT usage rates. However, as the study population was self-selected, restricted to women with MRS ≥5, and had a higher prevalence of prior HT use than the reference population, the observed difference in HT initiation may reflect differences in baseline characteristics or selection bias rather than an effect of menopause education.

Our findings are consistent with prior literature that examined the relationship between menopause education and HT initiation. A cross-sectional study identifying factors that influenced women's decisions to seek HT found that physician counseling, patient knowledge about HT, and employment status were significant predictors of treatment initiation (17). Similarly, a study examining nurse-assisted telephone counseling found that patient knowledge about menopause symptoms and treatment options increased significantly (*p* = 0.007) and treatment decisional conflict decreased significantly (*p* < 0.001) when comparing the baseline and endpoint survey data, with nurse-assisted counseling occurring between the two surveys. This further supports the premise that informed patients are more likely to engage with treatment decisions (18). These results highlight the importance of patient education and its role in menopause management, findings that are consistent with the current study which demonstrated that menopause education, regardless of format, was associated with higher HT initiation than observed in the reference population.

The absence of a significant difference between the emmii app and the educational pamphlet is also consistent with prior work comparing multiple education modalities. One study randomized women across three educational interventions with equivalent content: a brochure, a traditional lecture-discussion format, and an interactive format involving individualized risk assessment and values clarification (19). All three modalities were deemed to effectively communicate the risks and benefits of HT with no significant differences between groups. Notably, that study was limited to women with higher levels of education, which may have attenuated any differential effect between the individual modalities (19). Taken together, the literature suggests that the content and availability of menopause education is more consequential than its format.

A central gap in existing literature is the absence of a standardized, evidence-based framework for menopause education. While prior studies have consistently demonstrated that no single modality outperforms another, the field has not yet identified the optimal content of menopause education, independent of its delivery format (20). Future research might investigate differences in educational content, such as whether instruction focused specifically on symptom recognition vs. treatment options produces differential effects on patient knowledge and treatment uptake. Identifying the most effective content and framing for menopause education would be a meaningful step toward developing standardized educational resources that ensure all women have access to accurate, consistent menopause information and available treatment options.

Relatedly, participants in this cohort identified weight gain and diet, for example, as among the most prominent menopause-related healthcare needs. However, weight gain is not currently represented in the MRS. This discrepancy underscores the need for instruments that incorporate a metabolic domain, as currently widely used, validated instruments may not capture the full spectrum of symptoms women prioritize, potentially limiting the acknowledgment and recognition of these concerns in both clinical encounters and research. In this context, the consistently lower satisfaction ratings compared with participants' ratings of the importance of these healthcare needs suggest a potential gap between the concerns women prioritize and the care currently available to address them.

These findings have several implications for clinical practice. Women who received any structured menopause education initiated HT at significantly higher rates than those who received none, suggesting that access to accurate education at the point of care may matter more than its delivery format. Clinicians and healthcare systems may benefit from prioritizing the integration of menopause education into routine clinical care encounters, irrespective of format.

### Strengths and limitations

This study has several notable strengths. The randomized controlled design minimizes the risk of selection bias and strengthens causal inference regarding the effect of education modality on menopause treatment initiation. Menopause symptom severity, as measured by the MRS, did not differ significantly between groups, allowing any observed differences in outcomes to be attributed to the mode of education rather than to differences in symptom burden at enrollment.

There are limitations to this study that merit discussion. The study population was recruited exclusively from Mayo Clinic, which may limit the generalizability of findings to broader clinical settings. The sample was also limited in both racial and ethnic diversity, and educational diversity, which constrains the degree to which results can be extrapolated to more heterogeneous populations. Menopause status was not directly assessed, and eligibility was based on age and MRS score, which limits our ability to analyze outcomes across different stages of the menopause transition. While this study invited participation by several methods including email, patient portal message, or phone call, it is possible that these methods are not equally perceived among eligible participants of various backgrounds (21). Additionally, all participants in the study were insured, which may further limit applicability to women without consistent access to healthcare. Expanding approaches to obtain input from more heterogeneous populations will be valuable to better understand the menopause experiences in primary care for all women.

Participation bias must be considered as a possibility, potentially with women who elected to participate in this study being more symptomatic or having a heightened interest in the topic. In addition, unlike the study population, which was restricted to women with an MRS score ≥5, the reference population was identified based on age alone and MRS scores were not available. Therefore, it is possible that the lower HT prescribing rates in this group could be, at least in part, due to lower symptom burden or other differences in baseline characteristics. Survey completion also differed between groups, with a lower completion rate in the app group than in the control group. The longer survey administered to participants in the app group, which included additional questions about app satisfaction, may have contributed to this difference in completion rates and introduces the possibility of response bias in the survey findings. Lastly, the 30-day follow-up period may have been too short to capture treatment decisions that can take longer to develop.

## Conclusions

This study found that structured menopause education, regardless of delivery format, was associated with significantly higher rates of HT initiation compared to women who did not receive educational materials. Potential residual confounding, stemming in part from limited information about the menopause symptoms and educational needs of women in the reference population, limits causal interpretation. However, these findings highlight a potential opportunity to improve menopause education at the point of care, encompassing both patient-facing resources and clinician training to support informed, evidence-based conversations about treatment options. Future research should prioritize identifying the optimal content of menopause education as well as conducting larger studies in more diverse populations. Addressing these gaps will be essential to ensuring that all women have equitable access to the information they need to make informed decisions about their menopause care.

## Data Availability

The raw data supporting the conclusions of this article will be made available by the authors, without undue reservation.
