# Supplementary material for: A mobile app compared with an educational pamphlet on menopause hormone therapy initiation: a randomized pilot study
Source: Front Reprod Health. 2026 Sep 16;8:1952673. doi: 10.3389/frph.2026.1952673 (PMC13624152; doi:10.3389/frph.2026.1952673)
Supplement: Supplementary file 1 [file Supplementaryfile2.pdf]

MAYO CLINIC

Patient Education

Perimenopause and menopause

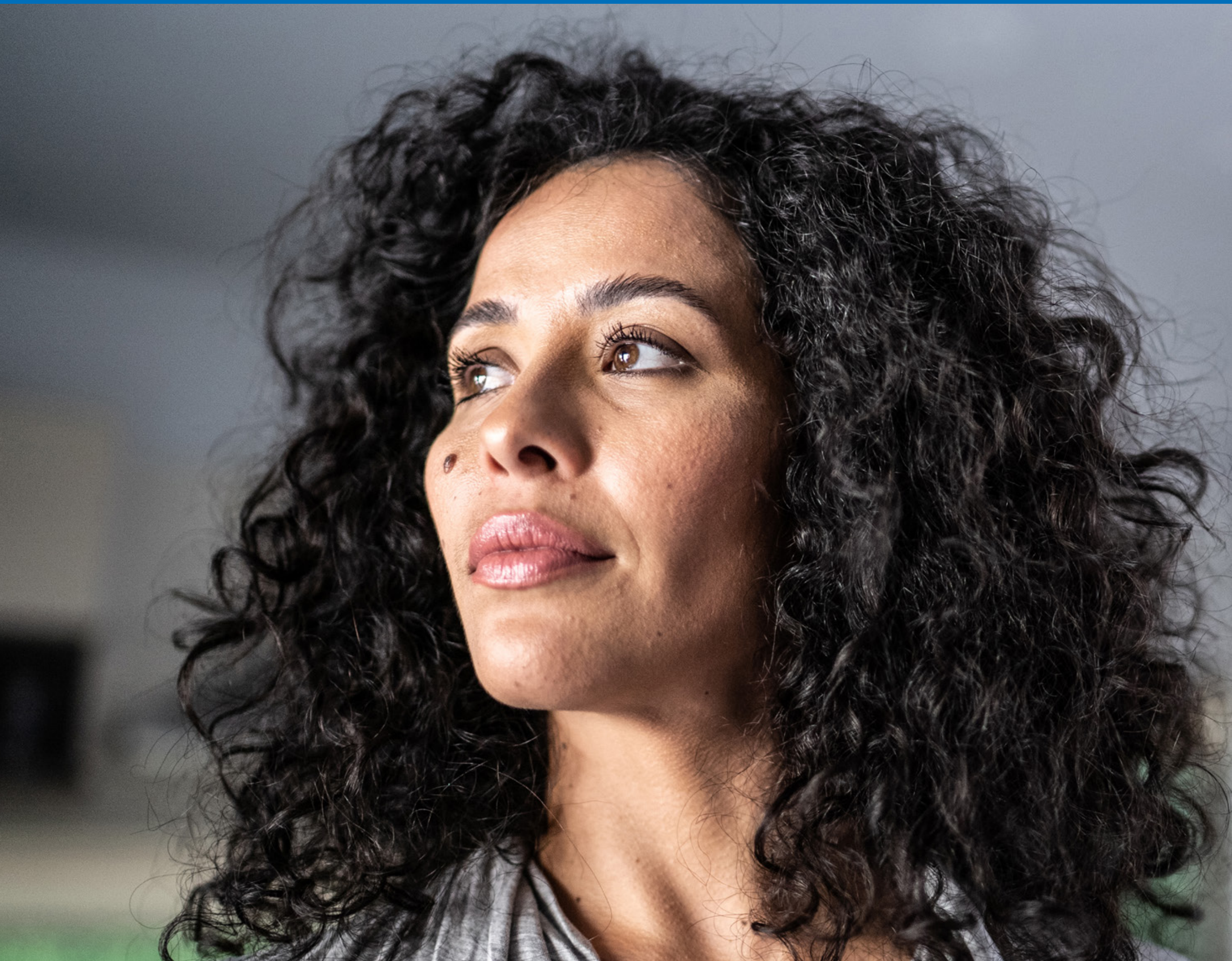

The process of menopause

Perimenopause and menopause are a natural part of aging. The process is a series of changes. It is not a single event. Changes start in your 30s or 40s. They may last into your 50s or 60s.

The transition from perimenopause to menopause

The time before menopause is called perimenopause. It can last months to several years or more. You reach menopause when you have gone 12 months in a row without a period. The perimenopause years and early years after menopause are the menopause transition.

During this time, hormone levels change unevenly. These changes often show up first as changes in your menstrual cycle. Other changes may include physical symptoms and cognitive changes. These are changes in thinking.

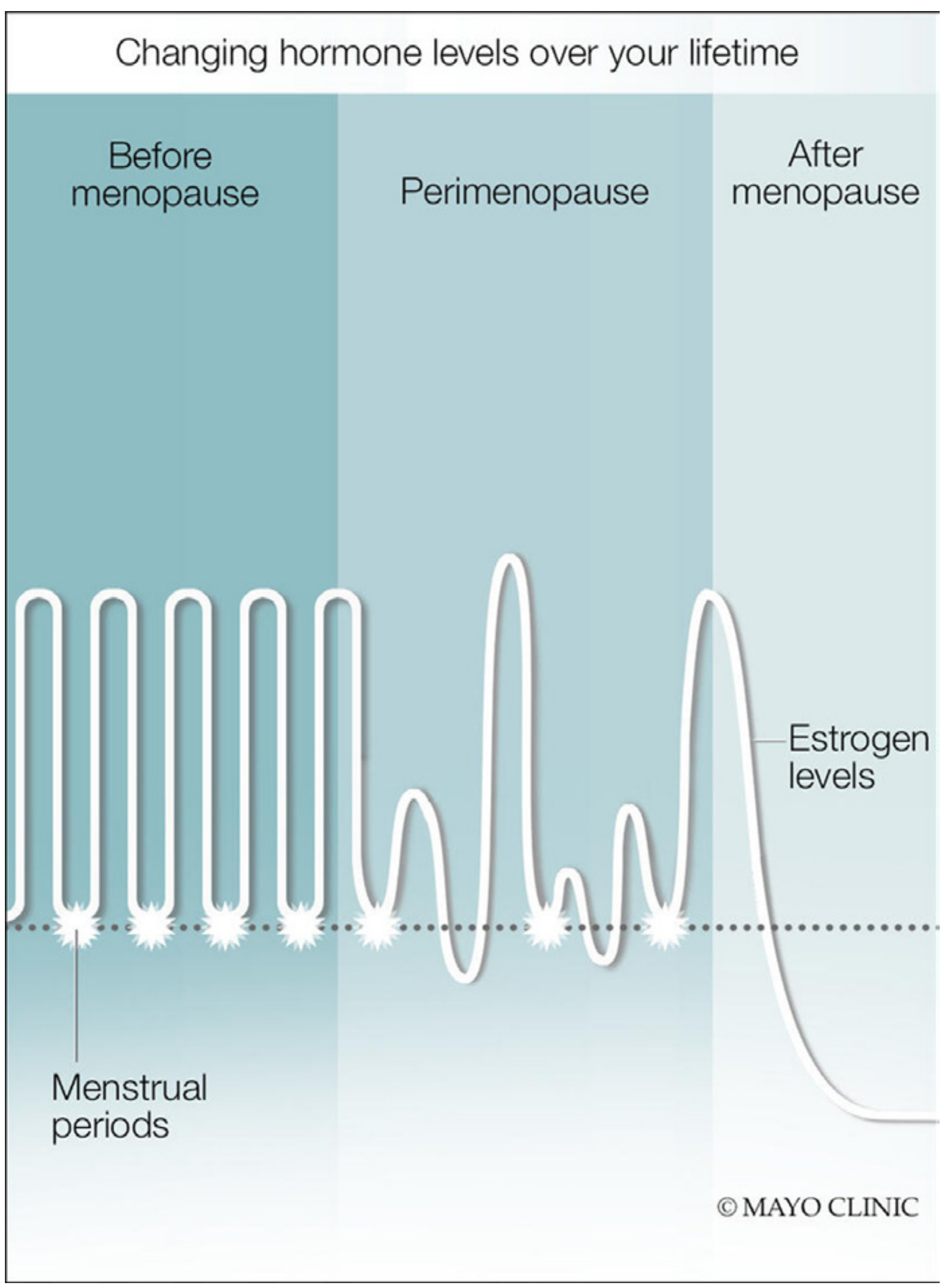

Changes in your periods during perimenopause

Changes in your menstrual cycle start in perimenopause because the ovaries do not release an egg every cycle. When an egg is not released, the cycle is referred to as an anovulatory cycle. This is what causes hormone levels to go up and down. Then menstrual cycles become irregular. Changes in the menstrual cycle are often the first sign that menopause is getting closer. You may notice one or more of the following changes in your menstrual cycle.

- Periods that are closer together. They happen more often than every 28 days.
- Periods that are farther apart.
- Physical symptoms such as cramping may be more painful or less painful than usual.
- Bleeding is heavier or lighter than usual.
- Bleeding lasts longer or is shorter than usual.

If you do not have regular periods, you may not notice these changes. You also may not notice changes if you:

- Take hormonal medicines, including birth control pills, hormone therapy or an intrauterine device (IUD).
- Have had a procedure to lessen bleeding such as a uterine ablation.
- Have had surgery to remove the uterus, called a hysterectomy.

Fertility changes during perimenopause

As your menstrual cycle becomes less regular, your ability to become pregnant lessens. But until you have gone a year without a period, you can still become pregnant.

- If you are not in menopause but wish to become pregnant and are having difficulty doing so, talk with your healthcare team.
- If you wish to avoid pregnancy, use a reliable form of birth control. Pregnancy is still possible until you have not had a period for a full year. Talk about birth control needs with your healthcare team.

Diagnosing perimenopause

Changes in your period are just one item your care team considers when diagnosing perimenopause. Your healthcare team may use other information to help find out what stage of perimenopause or menopause you may be in. This may include:

- Surgical history.
- Symptoms.
- Lab testing that may help identify other causes of symptoms but is not used to diagnose perimenopause.

The timing of menopause

Many people notice symptoms of perimenopause, such as hot flashes or sleep problems, in their 40s. Hormone changes can begin earlier, sometimes in the mid-30s. Most people reach menopause between the ages of 45 and 55.

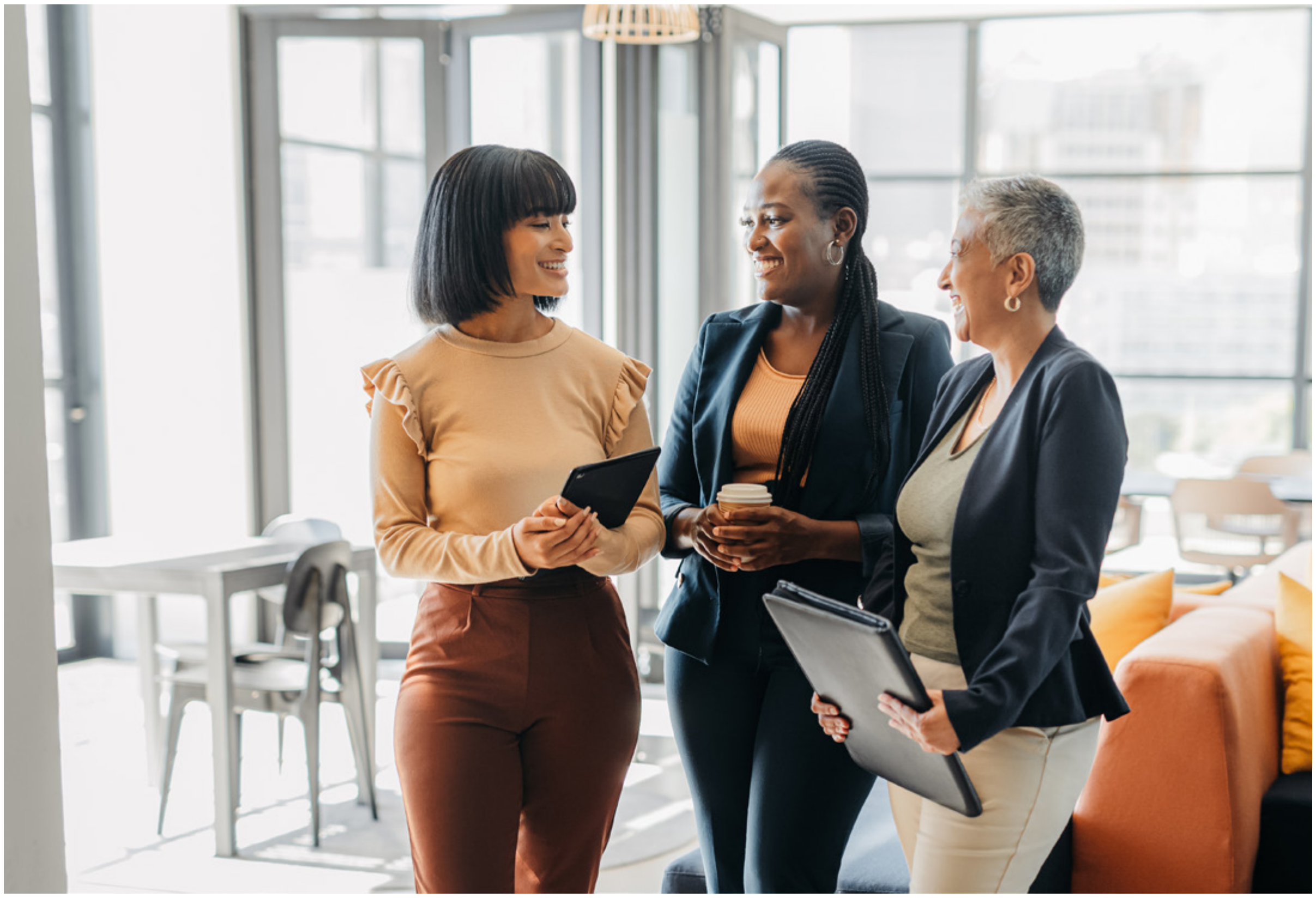

Menopause caused by surgery or medical treatment

Some medical treatments can cause menopause earlier than usual.

- **Removal of the ovaries.** This surgery is called oophorectomy. Menopause happens right away after this surgery.
- **Chemotherapy or pelvic radiation.** These treatments can damage the ovaries. That may cause temporary or permanent menopause.
- **Certain medicines.** Some treatments, such as hormone-blocking therapies used for cancer, can cause menopause while they are taken. Or they may cause permanent menopause.

A hysterectomy that removes only the uterus, not the ovaries, does not directly cause menopause. The ovaries continue to function and produce hormones even when menstrual bleeding no longer occurs after hysterectomy. A person who has had a hysterectomy will not have periods to track the menopause transition. But other symptoms still may develop.

Risks of early menopause or medically caused menopause

Menopause is considered early when it happens before age 45. Early menopause means less estrogen in the body for a longer time. This can increase the risk of some health concerns later in life. If menopause happens early, talk with your healthcare team about ways to protect your long-term health.

When to seek medical care for perimenopause or menopause

Most people do not need lab tests to confirm perimenopause or menopause. Because hormones move up and down widely in perimenopause, routine hormone testing is not advised. Menopause is confirmed when you have had no periods for one year.

Irregular periods are a common sign of perimenopause. See your healthcare team if you have:

- Very heavy bleeding. For example, you soak a pad or tampon every hour.
- Bleeding that lasts longer than 10 days.
- Periods that are more painful than usual and affect your daily life.
- Bleeding between periods.
- Bleeding after menopause. This is bleeding after you have gone 1 full year or longer without a period.
- Skipped periods when you are not in the typical age range for perimenopause.

Your healthcare team may ask about your symptoms, do an exam, and advise tests if needed.

Continue to see your healthcare team during perimenopause and after you reach menopause. Preventive care, including regular pelvic and breast exams, continues to be important. Some conditions linked to aging cannot be avoided. Other conditions can benefit from habit changes and medical therapies.

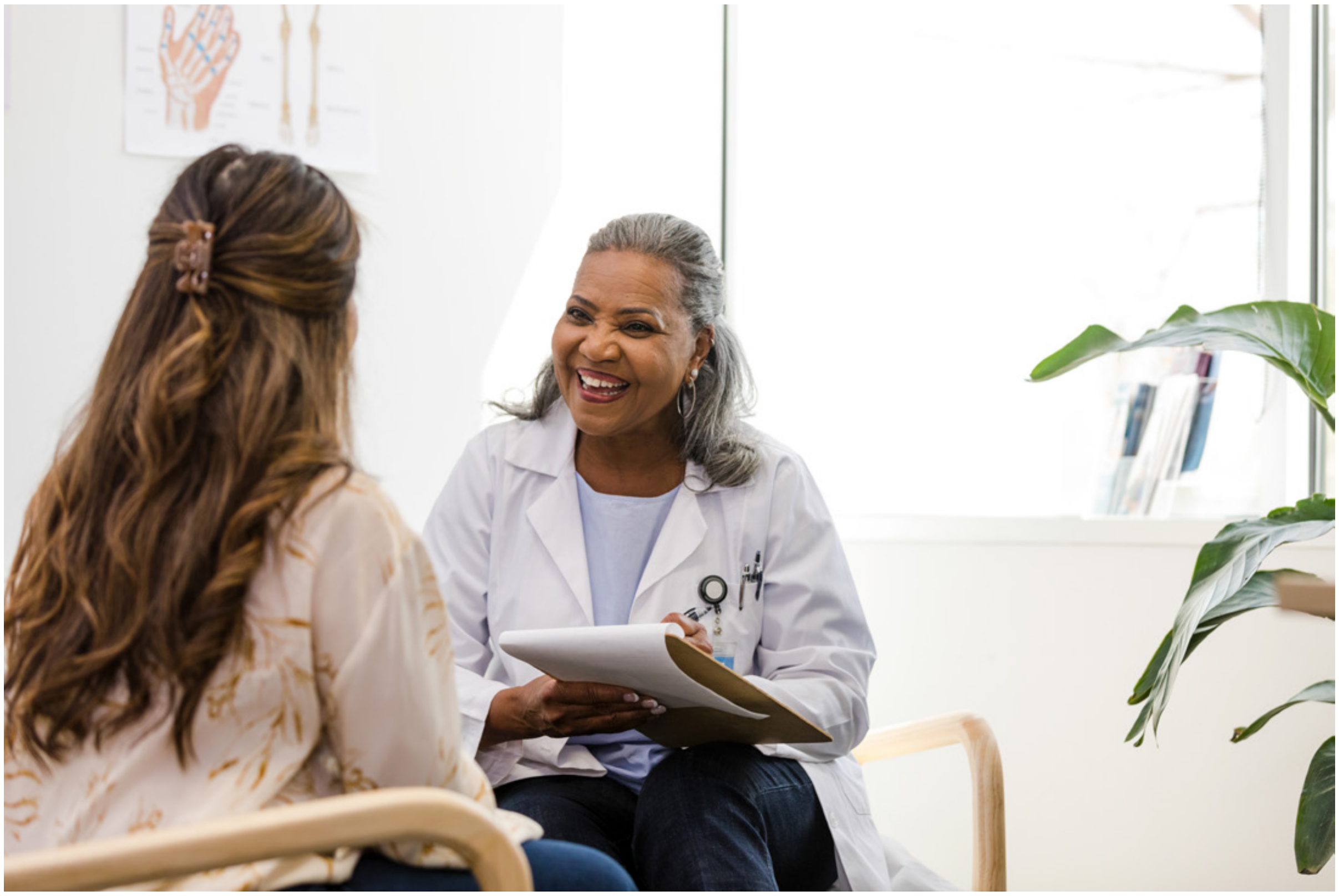

Tracking your menstrual cycle and symptoms during the menopause transition

It may help you to track your symptoms in a diary, a calendar, a period app or in another way that works for you. You can share this information with your healthcare team. Track periods, hot flashes and night sweats. Track sleep problems, vaginal dryness and mood changes. This way, the healthcare team can better understand your experience. Over time, team members can use this information to track whether any treatments work.

Symptoms of perimenopause and menopause

Symptoms during perimenopause and menopause vary widely. Some people have few or no symptoms. Others have symptoms that affect daily activities.

Hot flash symptoms during perimenopause and menopause

Hot flashes are the most common symptom of perimenopause. You may hear them called vasomotor symptoms. They are sudden sensations of heat. They often start in the chest and move to the neck and face. Sweating, flushing or chills may occur. You may sweat and as the sweat evaporates from your skin, you may feel chills. You also may feel weak and sometimes slightly faint. Your face may look flushed. Red blotches may appear on your chest, neck and arms.

The intensity, duration and frequency of hot flashes vary from person to person. Most hot flashes last from one minute to several minutes, although they can last longer. You may have them a few times a month or several times a day. They can occur at any time but often are worse at night. Some people find that certain things can cause a hot flash. These can include alcohol and smoking. They can include caffeine, hot beverages or hot temperatures. Some medicines and some medical conditions also can make hot flashes more noticeable.

Contact a healthcare professional if hot flashes affect your sleep or daily activities.

Sleep disturbance symptoms during perimenopause and menopause

Sleep problems are common during perimenopause and menopause. Nighttime hot flashes or sweating may disrupt sleep. Sleep problems also can occur without hot flashes. You may wake from a sound sleep with night sweats followed by feeling chilled. You may have difficulty falling back to sleep or getting deep, restful sleep.

Poor sleep can lead to irritability and mood changes. It can lead to brain fog.

Vaginal and lower urinary tract symptoms during perimenopause and menopause

Lower estrogen levels can cause the tissues of the vulva, vagina and urinary tract to become drier and thinner. Symptoms may include vulvovaginal dryness, burning and pain with sex. They also may include frequent urination or leaking urine.

Contact a healthcare professional if you have genital discomfort, bladder symptoms or pain with sex.

Body and skin symptoms during perimenopause and menopause

As you age, lower estrogen levels affect:

- Bone health and density. During the menopause transition, you may lose bone density at much faster rates than before. This can lead to low bone density, called osteopenia. Or it can lead to osteoporosis, where bones become thin and brittle. Both conditions put you at higher risk of fractures.
- Body composition. You may lose muscle while you gain fat around your belly.
- Collagen in your skin. This can make skin thinner and less elastic. It can result in wrinkles in your skin. You also may notice a loss of fullness in your breasts.

Even though your estrogen levels drop, your body continues to produce small amounts of testosterone. This can affect:

- Hair changes. Hair on the head may thin. Some people notice more coarse hair growth on the chin, upper lip, chest or abdomen.
- Adult acne may become worse.

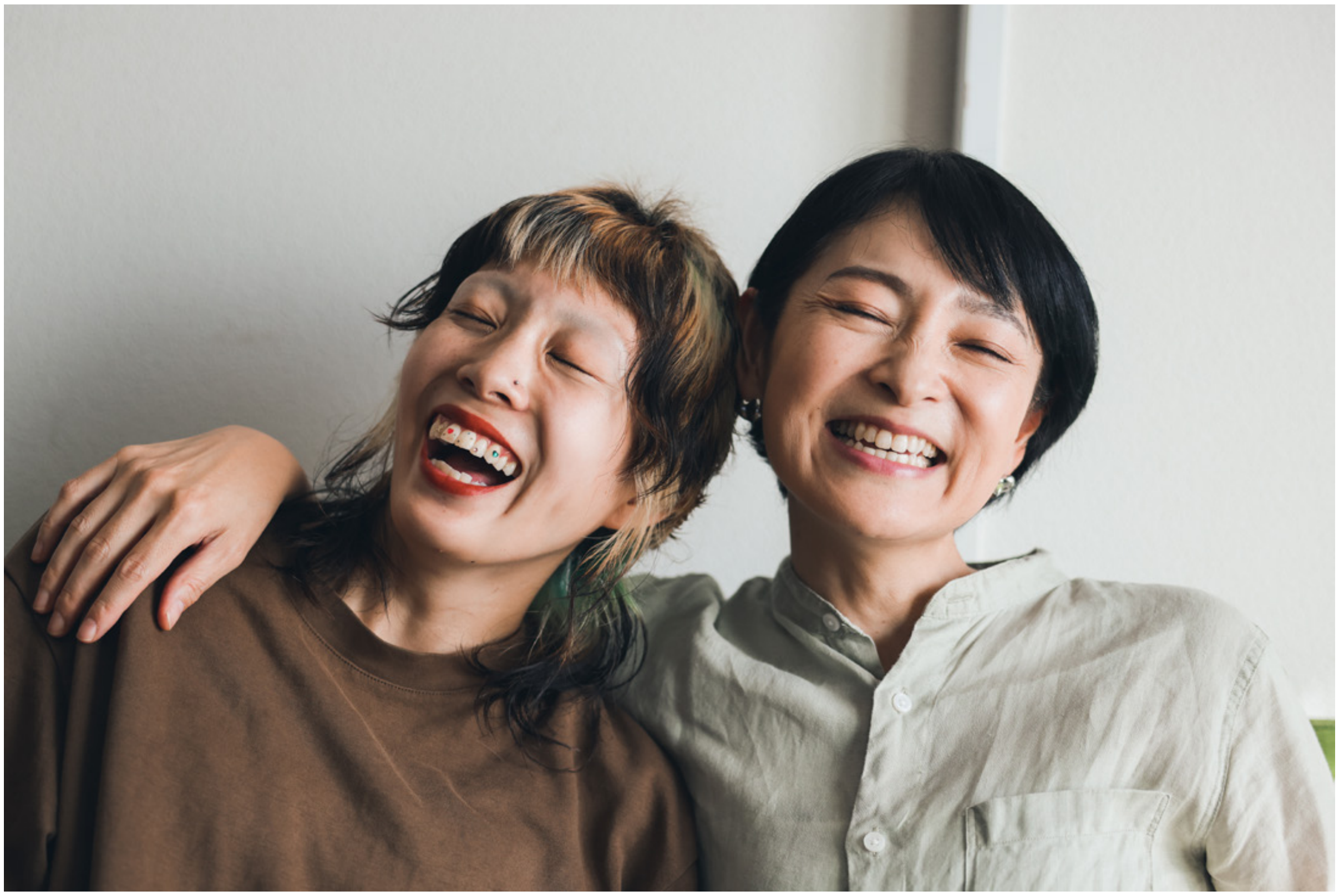

Mental changes and brain fog during perimenopause and menopause

Many people notice changes in their thinking during perimenopause. These changes are usually mild and temporary. They are not the same as developing dementia.

This often happens at the same time as changes in the menstrual cycle and other physical symptoms. Hormone changes, especially estrogen changes, may be linked to changes in parts of your brain involved in memory, attention and processing speed. Some people describe this as brain fog. Brain fog refers to more-frequent forgetfulness, trouble concentrating or difficulty finding words. Other physical symptoms that may be common during perimenopause can make thinking problems feel worse. They include symptoms such as sleep disruption, hot flashes, mood changes and stress.

Emotional changes during perimenopause and menopause

Some people have mood swings, depression, anxiety or fatigue as part of menopausal changes. But psychological symptoms may happen for many reasons, not just menopause. Stress, insomnia and life events tend to occur in this stage of adulthood. Examples include the illness or death of a parent. They include grown children leaving home or moving back. They also include career transitions and responsibilities. Perimenopause and menopause may worsen symptoms for people with a history of mood disorders associated with hormonal changes. They include premenstrual syndrome, postpartum depression or anxiety. They also include premenstrual dysphoric disorder (PMDD).

Sexual desire and fertility changes of perimenopause and menopause

Interest in sex can change at any point, including during midlife. For some people, interest in sex decreases with perimenopause and menopause. For others, it increases.

While some of the factors are hormonal, other important things can affect sexual desire. They include:

- Availability of a partner.
- A partner's sexual function.
- Medical conditions of either you or a partner.
- Medicines for either you or a partner.
- Family or relationship stressors.
- Other life issues. Stress can lessen sexual desire at any time.

Easing menopause symptoms and caring for yourself

Although menopause is associated with hormonal, physical and psychosocial changes in your life, it isn't the end of your life or your sexuality. You may spend as much as a third or more of your life after menopause. It is the start of a new chapter.

More is known about menopause now than ever before. You have options to ease symptoms and lessen complications. You have options to improve your health during this important phase of your life.

You may not need treatment during menopause. But you can treat symptoms if they bother you. You can work with your healthcare team to prevent or address chronic conditions that may occur during the menopausal years.

Treating hot flashes during perimenopause and menopause

- Wear lightweight layers or breathable fabrics to help regulate body temperature.
- Identify and avoid triggers that can increase hot flashes. Common triggers include warm environments, hot drinks and spicy foods. They include alcohol, caffeine and smoking.
- Try deep breathing. It can lessen the intensity of hot flashes for some people.

Talk to your healthcare team if these suggestions do not give enough relief from hot flashes. Discuss the use of hormone therapy. Find out about options that are not hormonal. Find out about prescription therapies to ease hot flashes.

Treating sleep issues during perimenopause

Ensure good sleep habits, also called sleep hygiene.

- Sleep in a cool room, keep a window open or use a ceiling fan. Keep a small fan by your bed.
- Try light, cooling sleepwear or perspiration-wicking fabric, sheets and blankets. Instead of one heavy cover, put a few thin blankets on the bed. Remove them as needed.
- Do not eat large meals or drink alcohol before bed.
- Try not to eat or drink anything with caffeine after noon.

Talk with your healthcare team if you have disturbed sleep.

Treating vulvovaginal changes during perimenopause

- Try different types of sexual stimulation or touch before intercourse.
- Use a water-based or silicone-based lubricant to help lubricate the tissues while you engage in any sexual activity.
- Use vaginal moisturizers to help moisturize the genital tissues.
- Avoid irritating chemicals such as products with fragrances, parabens and glycerin.
- Talk with your healthcare team about vaginal estrogen therapy. This is the most effective treatment for symptoms like vulvovaginal dryness and pain with intercourse. Vaginal estrogen therapy comes in several forms. They include cream, tablets or a ring that is inserted into the vagina. If you cannot or prefer not to use estrogen, other options are available.

Talk with your healthcare team if you have vaginal itching, dryness, bleeding or unusual discharge.

Treating bladder changes during perimenopause

- Limit fluid intake a few hours before bedtime.
- Avoid common bladder irritants. They include caffeine, citrus and carbonated beverages. They include high-dose vitamin C and B vitamin supplements. They also include alcohol and artificial sweeteners.
- Talk with a pelvic floor physical therapist or a member of your care team if you have bladder concerns.
- Talk with your healthcare team about vaginal estrogen therapy. This is a common preventive therapy after menopause for people with recurrent urinary tract infections. Vaginal estrogen therapy comes in several forms, such as cream, tablets or a ring that is inserted into the vagina.

Contact your healthcare team if you have continual discomfort with urination or any other bladder symptoms.

Treating body and skin changes during perimenopause

- Eat healthy foods like vegetables, fruits and whole grains. Eat lean dairy products and lean meats. Eat fish and plant-based proteins. Eating plenty of fiber is important for health. Try for at least 25 grams of fiber each day. Eating enough protein is important to maintain muscle. There is no specific diet that is advised for those going through perimenopause.
- Add cardiovascular exercise, strength training and flexibility training into your weekly schedule.
- Keep your body hydrated and well rested.
- Apply moisturizer to your skin after you bathe.
- Apply sunscreen before you go outside. Reapply sunscreen every 80 minutes while in the sun.

Treating emotional and thinking changes during perimenopause

- Treat physical symptoms. In many cases, brain fog and other memory or concentration issues are made worse by other perimenopause symptoms. Treating these symptoms can help improve thinking issues too.
- Strive for a healthy lifestyle. Get plenty of sleep and exercise. Eat healthy foods. Research has shown that even short periods of light exercise are beneficial for improving mood.
- Try to lessen the physical and psychological effects of stress. Take part in exercise. Meditate. Do yoga. Learn about biofeedback. Keep a journal. Do positive visualization or massage.
- Use your time and energy wisely. Try to pace yourself at home. Do not overcommit at your place of worship and in the community. Remember to save some time for you. A healthy balance between work, caring for others and taking care of yourself is important to your emotional well-being.
- Find support. Share concerns with family members or friends. Talk with others going through a similar experience. Join a support group.

Treating depression and anxiety during perimenopause

Menopause alone does not cause mental health concerns. But people who are at increased risk of depression or anxiety may experience more of these symptoms during menopause. If you think you have symptoms of depression or anxiety, talk to your healthcare team. Treatment for depression and anxiety is effective. It can help you enjoy life again.

Practicing healthy habits and taking care of yourself during perimenopause

- Decide what’s most important in your life and base decisions on your values.
- Limit or eliminate caffeine and alcohol from your diet.
- Stop smoking.
- Try new activities. Try a class, start a hobby. Volunteer or share your skills through community activities.

Improving your sexual health during perimenopause

Talk with a partner about your relationship. Improved communication may help increase feelings of emotional closeness and intimacy. Try to add more romance into your life.

- Do things that add connection to your relationship. Hold hands, listen to and help each other. Go for walks together or dance together. Tell each other when you appreciate the other. Write a note or simply enjoy quiet time together.
- Reflect on your relationship and the wonderful times. Remember how you overcame the challenging times together.
- Experiment with ways to increase your sexual satisfaction and desire. This may include trying new types of stimulation and touch. You also can try lubricants and relaxation techniques.

If you have sexual concerns, talk with your healthcare team or seek referral to a licensed sex therapist.

Preventing bone density loss during perimenopause

- Get at least 1,200 milligrams of calcium each day. If you do not reach this goal with your food, take calcium supplements with a meal or snack.
- Get enough vitamin D for good bone health. Sources of vitamin D include sunlight exposure, vitamin D-fortified milk, multivitamins and some vitamin D-containing calcium supplements. Your calcium and vitamin D recommendations may be slightly different if you already have osteoporosis or osteopenia.
- Take part in regular weight-bearing exercises to keep your bones strong.
- Consider a bone density test to check for osteoporosis or osteopenia.

Talk with your healthcare team about bone-building medicines and hormone therapy to help prevent bone loss.

If you have low bone density or osteoporosis and are not already on treatment, talk with your healthcare team about treatment options.

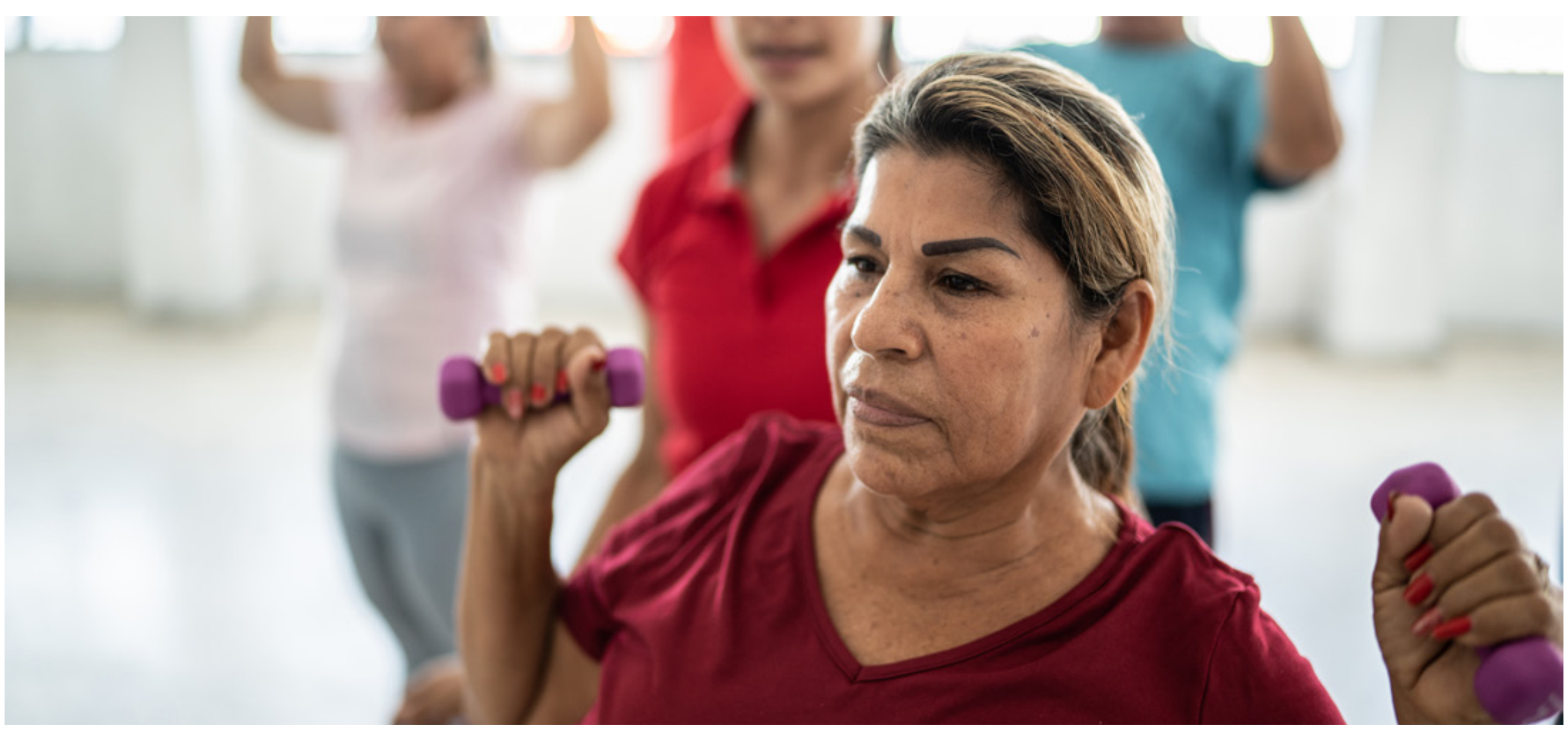

Health conditions linked to perimenopause and menopause

Several chronic medical conditions tend to increase after menopause. By becoming aware of the following conditions, you can take steps to help lessen your risk.

Cardiovascular disease during perimenopause and menopause

The risk of cardiovascular disease increases with age and as your estrogen levels decrease. Coronary artery disease is the leading cause of death. Yet you can do a great deal to lessen your risk of heart disease.

- Stop smoking.
- Limit or eliminate alcohol intake.
- Lower high blood pressure.
- Get regular aerobic exercise.
- Eat a heart-healthy diet.
- Get treatment for high cholesterol and diabetes.

Ask your healthcare team for a specific prevention and monitoring plan. Hormone therapy is not usually recommended to prevent cardiovascular disease. There are many other factors that determine cardiovascular health as well as other factors that go into deciding if hormone therapy is right for you.

Urinary incontinence during perimenopause and menopause

Lower estrogen levels can affect the tissues of the bladder and urethra. This may contribute to leakage, called urinary incontinence. It can contribute to a feeling of urgency or difficulty getting to the bathroom in time. Pelvic floor muscle exercises and pelvic floor physical therapy may help lessen leakage. You may benefit from making changes in your fluid intake, frequency of urinating or medicines. Talk to your healthcare team about other treatments that are available.

Weight changes during perimenopause and menopause

Changes in metabolism and hormone levels after menopause may affect body weight and fat distribution. Weight gain can happen even without changes in eating habits. You may need to eat fewer calories a day. And you may need to exercise more just to maintain your current weight.

Strength training to maintain and build muscle mass helps with long-term weight management. Menopause hormone therapy is not used for weight loss, as it has not been shown to help.

Enjoying life after menopause

Perimenopause and menopause are natural life stages. Understanding the changes that happen during these stages and their effects on the body can help you make informed health decisions. You may be better prepared to deal with symptoms and life changes. There are healthcare teams who can support you through this transition. Don't let menopause stop you from enjoying this next stage of life.

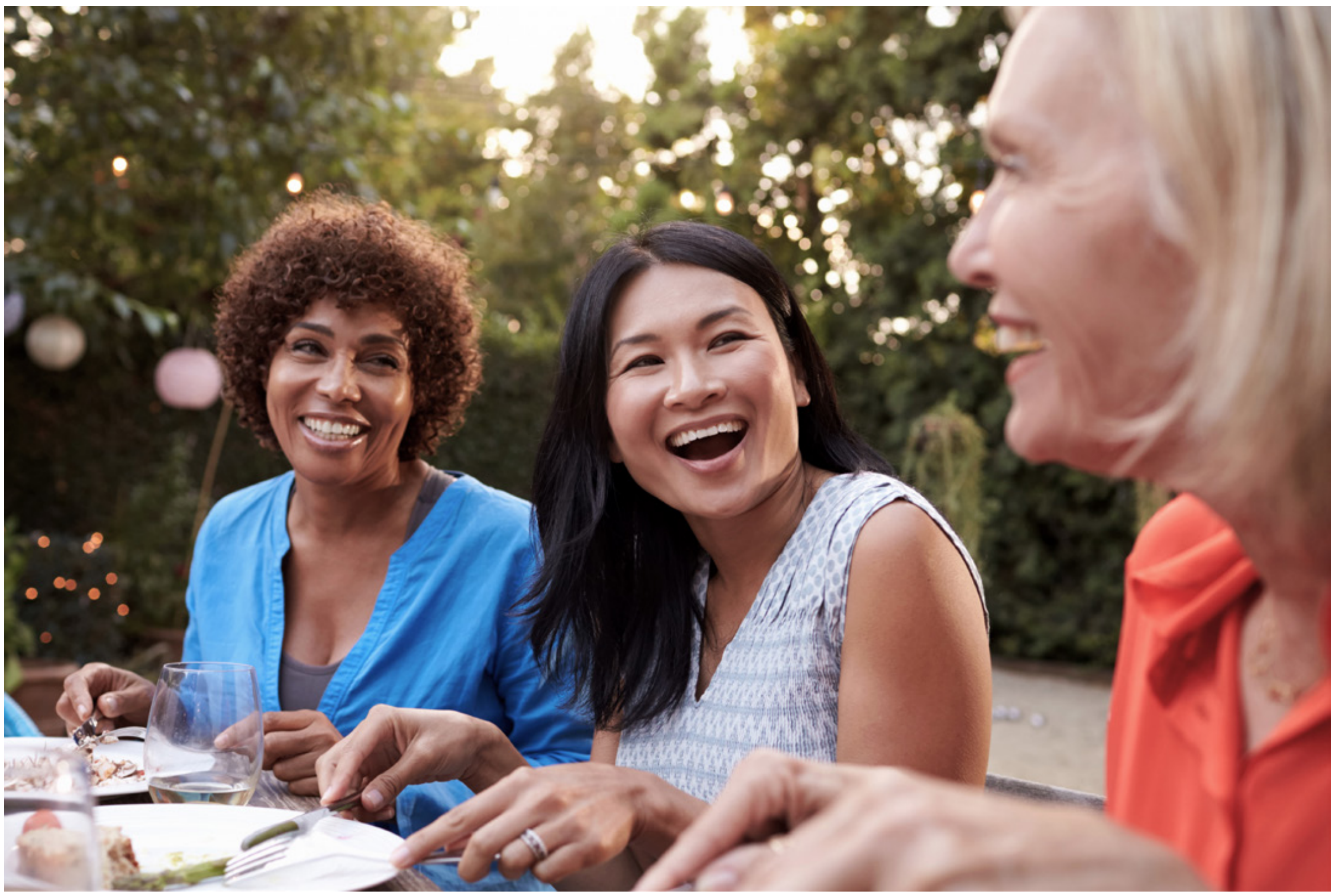

Notes

Notes

Notes

Barbara Woodward Lips
PATIENT EDUCATION CENTER

Mrs. Lips, a resident of San Antonio, Texas, was a loyal patient of Mayo Clinic for more than 40 years. She was a self-made business leader who significantly expanded her family's activities in oil, gas and ranching, even as she assembled a museum-quality collection of antiques and fine art. She was best known by Mayo staff for her patient advocacy and support. Upon her death in 1995, Mrs. Lips paid the ultimate compliment by leaving her entire estate to Mayo Clinic.

Mrs. Lips had a profound appreciation for the care she received at Mayo Clinic. By naming the Barbara Woodward Lips Patient Education Center, Mayo Clinic honors her generosity, her love of learning, her belief in patient empowerment, and her dedication to high-quality care.

> This information is for your education only. It does not replace medical advice, diagnosis or treatment. New medical research or practices may change this information. If you have questions, talk with a member of your healthcare team.

Images, if used, are copyright MFMER,
Getty Images or Shutterstock, or approved for use.

mayoclinic.org

©2026 Mayo Foundation for Medical Education and Research. All rights reserved. MAYO, MAYO CLINIC and the triple-shield Mayo logo are trademarks and service marks of MFMER.

MC5544rev0426
